# Supplementary figures and images for: Transcriptome Analysis of the Effect of Nickel on Lipid Metabolism in Mouse Kidney
Source: Biology (Basel). 2024 Aug 24;13(9):655. doi: 10.3390/biology13090655 (PMC11429462; doi:10.3390/biology13090655)

GAPDH

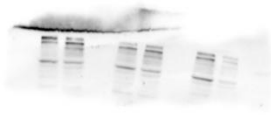

AMPK

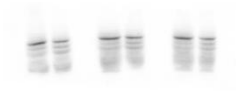

FASN

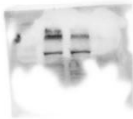

GAPDH

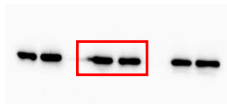

FASN

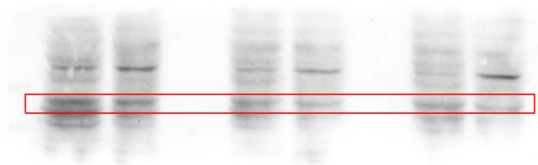

GAPDH

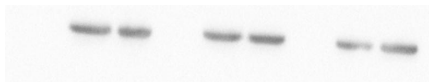

Supplement: Supplementary file 1 [file biology-13-00655-s001.zip › WB.pdf]
